# SEISMICgraph: a web-based tool for RNA structure data visualization

**DOI:** 10.1093/nar/gkaf701

**Published:** 2025-07-31

**Authors:** Federico Fuchs Wightman, Grant Yang, Yves J Martin des Taillades, Casper L’Esperance-Kerckhoff, Scott Grote, Matthew F Allan, Daniel Herschlag, Silvi Rouskin, Lauren D Hagler

**Affiliations:** Department of Microbiology, Harvard School of Medicine, Boston, MA 02115, United States; Department of Microbiology, Harvard School of Medicine, Boston, MA 02115, United States; Department of Biochemistry, Stanford University, Stanford, CA 94305, United States; Department of Microbiology, Harvard School of Medicine, Boston, MA 02115, United States; Department of Microbiology, Harvard School of Medicine, Boston, MA 02115, United States; Department of Microbiology, Harvard School of Medicine, Boston, MA 02115, United States; Department of Biochemistry, Stanford University, Stanford, CA 94305, United States; Department of Microbiology, Harvard School of Medicine, Boston, MA 02115, United States; Department of Biochemistry, Stanford University, Stanford, CA 94305, United States

## Abstract

In recent years, RNA has been increasingly recognized for its essential roles in biology, functioning not only as a carrier of genetic information but also as a dynamic regulator of gene expression through its interactions with other RNAs, proteins, and itself. Advances in chemical probing techniques have significantly enhanced our ability to identify RNA secondary structures and understand their regulatory roles. These developments, alongside improvements in experimental design and data processing, have greatly increased the resolution and throughput of structural analyses. Here, we introduce SEISMICgraph, a web-based tool designed to support RNA structure research by offering data visualization and analysis capabilities for a variety of chemical probing modalities. SEISMICgraph enables simultaneous comparison of data across different sequences and experimental conditions through a user-friendly interface that requires no programming expertise. We demonstrate its utility by investigating known and putative riboswitches and exploring how RNA modifications influence their structure and binding. SEISMICgraph’s ability to rapidly visualize adenine-dependent structural changes and assess the impact of pseudouridylation on these transitions provides novel insights and establishes a roadmap for numerous future applications.

## Graphical Abstract

**Graphical Abstract:**
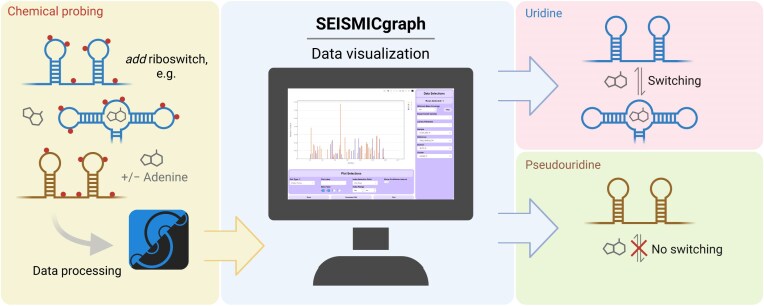

## Introduction

Since its initial discovery as an intermediary of genetic information, RNA has been recognized for its diverse regulatory roles, including the control of transcription, translation, splice site selection, and RNA degradation [1, 2]. These functions are achieved through RNA’s ability to bind proteins and fold into complex structures. The discovery of novel RNA interactions and their biological function has been driven and supported by the development of numerous techniques, each with specific advantages and limitations [3–7].

Chemical probing methods provide detailed insights into RNA base-pairing, folding, and interactions with biomolecules such as proteins and ligands at single-nucleotide resolution. These techniques use small molecules such as dimethyl sulfate (DMS) and SHAPE reagents [e.g. 1-methyl-7-nitroisatoic anhydride (1M7)] to covalently modify RNA at chemically accessible sites [8–11]. The differential chemical reactivity between nucleotides is used to distinguish between folded and unfolded regions of a given RNA structure [12–14]. DMS methylates the Watson–Crick–Franklin face of solvent-exposed and unpaired A and C bases, while SHAPE reagents selectively acylate the 2′-hydroxyl group of bases in non-base-paired regions that can attain the reactive configuration [15–17]. Both DMS and SHAPE techniques can be combined with mutational profiling (MaP), where chemically modified residues are converted into mutations during reverse transcription to enable the generation of high-resolution structural maps across RNA molecules [13, 14, 18]. These methods are highly adaptable and can be employed to study how environmental conditions impact RNA interactions, allowing comparisons between *in vitro* and cellular contexts and providing new insights into the relationship between RNA sequence, structure, and function.

As chemical probing methods have become more widely adopted, several computational tools have been developed to analyze the resulting sequencing data. These include DREEM [19], RNA Framework [20], ShapeMapper 2 [21], and the more recent SEISMIC-RNA [22]. These tools facilitate the alignment of sequencing reads to reference sequences, the identification and quantification of mutations, and the generation of mutation profiles for each reference. The data generated through these analyses can be further used to test hypothesis-driven models and draw biophysical or biological conclusions, including the generation of secondary structure models and the assessment of the effects of mutations or environmental changes. Despite their utility, these tools typically require users to have at least basic programming skills to perform quality control assessments, visualize data, and compare samples, posing challenges for those less familiar with bioinformatics.

To overcome these limitations and allow the broader community to effectively use these approaches, we introduce SEISMICgraph, a web-based platform. SEISMICgraph is designed to streamline and standardize the visualization and analysis of chemical probing data. It was validated using datasets from DMS-MaPseq experiments involving well-characterized RNA structures. The application provides an accessible framework for generating plots and analyzing raw chemical probing data from RNA libraries. SEISMICgraph enables researchers to produce standard visualizations of chemical probing data, making the interpretation of results more accessible and interpretable, while also facilitating broad use in the scientific community. SEISMICgraph is available at https://www.seismicrna.org.

## Materials and methods

### Library design

The library used for the initial analysis contained 48 known RNA structures. We chose to use a short RNA (170 nt) design to mitigate several known limitations of library experiments (i.e. massively parallel reporter assays). First, common primers were used for each sequence in the library to ensure non-biased amplification. Next, unstructured flanking sequences were used to make each library member the same length, reducing PCR bias. Finally, barcodes were included to ensure accurate assignment to the designed reference. The full sequence structure of the libraries used in this work is shown in Fig. 2A. Designed RNA libraries were ordered as DNA oligo pools from Integrated DNA Technologies (IDT). Library amplification, *in vitro* transcription, DMS-MaPseq, and data processing were performed as outlined below with minor changes. Specifically, DMS reactions varied in magnesium (MgCl_2_) concentration (0, 1, and 10 mM), while keeping the RNA input (10 μg), buffer (150 mM potassium cacodylate, pH 7.4), DMS concentration (5% v/v), reaction time (5 min), and temperature (37°C) constant. One reference (*add* aptamer domain) and section (variable region, bases 40–112) were chosen from the library to help visualize the plot types in Fig. 2.

A second library was designed with the same general strategy (Fig. 3A) to probe the structures of known and putative RNA riboswitches. We retrieved from the RiboswitchDB database [23] 29 predicted Mg^2+^-dependent riboswitches and 30 proposed to respond to purines. We included the *add* riboswitch from *Vibrio vulnificus* as a positive control. The total library encompasses 81 sequences, including above mentioned putative riboswitches and other well-known structures such as the HIV frameshifting stimulation element (FSE) (see Supplementary Table S1). To enable pooled chemical probing with DMS-MaPseq, we designed the library to include the following: (i) Common forward and reverse primer sequences (forward primer: 5′- TTAAACCGGCCAACATACC -3′; reverse primer: 5′- TCGAAAGGAACGAGTAGCG -3′). (ii) Variable length flanking sequences to make all the constructs of equal length. Flanking sequences were generated by randomly shuffling the order of Cs and Us. Sequences were filtered for consecutive stretches of Cs or Us longer than 3 nucleotides and were regenerated for these cases. (iii) Unique barcodes were added to each sequence for later demultiplexing (Hamming distance >3), which was required because of similarities between several library sequences and to identify parent sequences after DMS modification (Figs 2A and 3A). In combination, these features increase sequencing read depth and quality, by decreasing RT and/or PCR biases (thereby giving more uniform reads of all library members) and by unambiguously assigning very similar sequences in the library pool. The complete sequences of the 81-member library are given in Supplementary Table S1. The DNA library was obtained as an oligo pool from IDT.

### DMS-MaPseq

The DNA library was first amplified via PCR, adding a T7 promoter sequence ahead of the forward primer (5′- TAATACGACTCACTATAG -3′), using the Q5^®^ High-Fidelity 2X Master Mix (NEB, Cat #M0492L) and the following program: 98°C for 2 min; 6 cycles of 98°C for 10 s, 63°C for 30 s, and 72°C for 30 s; a 2 min final extension at 72°C; and final hold at 4°C (for long-term storage, stored at −20°C). The annealing temperature was calculated using the NEB’s Tm calculator (https://tmcalculator.neb.com/#!/main). The reaction was carried out in a final volume of 50 μl, adding 2.5 μl of each primer (10 μM) and 1 μl of the oligo pool (0.1 pmol/μl). The cycle number was optimized by screening increasing numbers of cycles until nonspecific amplification was observed. To obtain the necessary yield of PCR product for the downstream applications, several reactions were run in parallel, pooled, and purified using a Zymo DNA Clean & Concentrator Kit (Cat. D4004).

*In vitro* transcription was performed using the T7 MEGAshortscript kit (Thermo Fisher, Cat. AM1354), starting with 200 ng of the amplified library. Twenty microliter reactions were incubated for 2–3 h, followed by DNase treatment for 15 min. For pseudouridine experiments, UTP was replaced with 75 mM ΨTP or m1ΨTP. A yield of 30–50 μg was obtained per reaction. The RNAs were purified using the Zymo RNA Large Sample Clean-Up Kit (Cat. R1017). For chemical probing, 5–10 μg of the purified RNAs were diluted in 10 μl of nuclease-free water, denatured for 1 min at 95°C, and placed on ice for 2 min. The RNA samples were refolded in 300 mM sodium cacodylate, pH 7.2, 6 mM MgCl_2_, and optionally 5 mM adenine (or an equivalent volume of DMSO as a control). Refolding buffer (85 μl) was added to the RNAs, which were then incubated at 37°C for 20 min in a thermomixer with a heated lid. Afterward, 5 μl of DMS was added to the RNA solution and the reaction was allowed to proceed at 37°C for 5 min with 800 rpm shaking in a thermomixer. The reaction was quenched by adding 60 μl of β-mercaptoethanol. The DMS-treated RNAs were purified using the Zymo RNA Clean & Concentrator-5 Kit (Cat. R1013). As recoveries can be as low as 10%–20%, increasing the initial quantity of RNA per reaction is sometimes helpful.

The purified RNAs were reverse transcribed using the Induro Reverse Transcriptase (NEB, #M0681), as described previously with minor modifications [24]. The reaction was performed as follows: 500–1000 ng of RNA, 1 μl of the reverse primer at 10 μM (sequence: 5′ TCGAAAGGAACGAGTAGCG -3′), 1 μl of 10 mM dNTPs, 0.2 μl of RNase inhibitor, murine (40 U/μl), 4 μl of the 5× Induro RT buffer, 1 μl of Induro reverse transcriptase (200 U/μl), and nuclease-free water up to 20 μl; the program was set for 30 min at 60°C, followed by the addition of 1 μl of 4 M NaOH for 3 min at 95°C. The complementary DNAs were purified using the Zymo Oligo Clean & Concentrator Kit (Cat# D4060) and gave a yield of ∼250–500 ng. We then amplified the constructs via PCR using the Q5^®^ High-Fidelity 2X Master Mix (NEB, Cat #M0492L) in a final volume of 50 μl and the following program: 98°C for 2 min, 15 cycles of 98°C for 10 s, 63°C for 30 s, and 72°C for 30 s, with a 2-min final extension at 72°C and a final hold at 4°C.

Samples were indexed using NEBNext Ultra II DNA Library Prep Kit for Illumina (#E7645S/L), following the manufacturer’s instructions, and sequenced on an Illumina NextSeq 1000 using a P1 (2 × 150 read) cartridge (Cat# 20050264).

### SEISMIC-RNA data processing

The first round of demultiplexing (separating the reads corresponding to each sample by their library index) was automatically performed by the Illumina platform BaseSpace (BCL-Convert 2.4.0) upon completion of the sequencing run. The FASTQ files were processed using SEISMIC-RNA (version 0.14.1) [22]. Briefly, SEISMIC-RNA demultiplexes based on the barcode of a given reference, aligns reads to the known sequence, and quantifies the number of mutations on each read at each position. The function *wf* (for workflow) was executed, including the option *-x* for 2 separate FASTQ files of paired-end reads; *--demult-on* for the second round of demultiplexing (a table with the barcode positions must be provided as well as a FASTA with the references, see Supplementary Table S1); *--sections-file* to annotate parts of the library sequence, including primers, flanking sequence, region of interest, and barcode; and *--export* for exporting a *json* file that can be provided to SEISMICgraph. Data were analyzed in SEISMICgraph. The filtered data for each plot were downloaded from SEISMICgraph and replotted in GraphPad Prism 10 to increase the resolution for publication.

## Results

### SEISMICgraph: a web-based analysis tool

**Figure 1.**
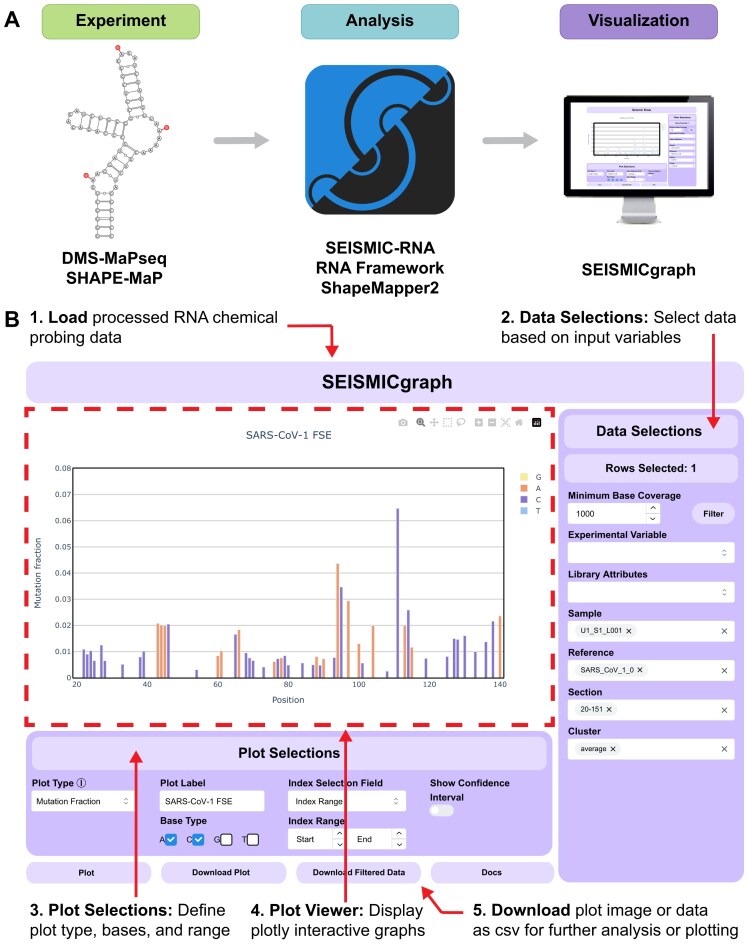
SEISMICgraph. (**A**) To study RNA secondary structures, DMS-MaPseq or SHAPE-MaP is performed by chemically modifying unstructured regions of RNA depicted here with red circles. Modifications are converted to mutations during reverse transcription polymerase chain reaction (RT-PCR) and read out by next-generation sequencing. The sequencing data from these experiments is then analyzed with SEISMIC-RNA, RNA Framework, or ShapeMapper 2 before being visualized with SEISMICgraph (see also Supplementary Fig. S1). (**B**) Overview of the SEISMICgraph interface.

We developed SEISMICgraph (Fig. 1 and Supplementary Fig. S1) as a web-based application to address the need for rapid and simple visualization of chemical probing data, without requiring programming skills. SEISMIC-RNA is a data processing algorithm that takes RNA-sequencing files from DMS-MaPseq or other chemical probing experiments and generates mutation profiles by calculating the mutation fraction at each nucleotide position. In contrast, SEISMICgraph is designed to take these mutation profiles as input and facilitate downstream visualization and analysis, helping users derive biological or biophysical insights. SEISMICgraph consists of three main parts: (i) a front-end user interface through which the user uploads data, makes selections, and requests plots; (ii) a backend server that handles these requests and responds to them; and (iii) a Python package (seismic-graph) that runs on the backend, processing uploads and generating plots.

Users can upload data from multiple experiments at once, up to 32 MB total. For datasets pre-processed with RNA Framework or SHAPEMapper 2, SEISMICgraph automatically converts them into SEISMIC-RNA format on upload. For details on how the data is structured, transformed, and organized, refer to the “Input data structure” section in Supplementary data.

#### User interface

The user interface is separated into three main sections (Fig. 1B): Data selections, Plot selections, and the Plot viewer.

In the Data selections panel, users choose a subset of the uploaded data to plot. Certain plot types require only one row to be selected at a time, while others require two or more rows to be selected. Additional filtering options are available depending on the data selected, with more detailed information provided in the “Data selection options” section in Supplementary data.

**Figure 2.**
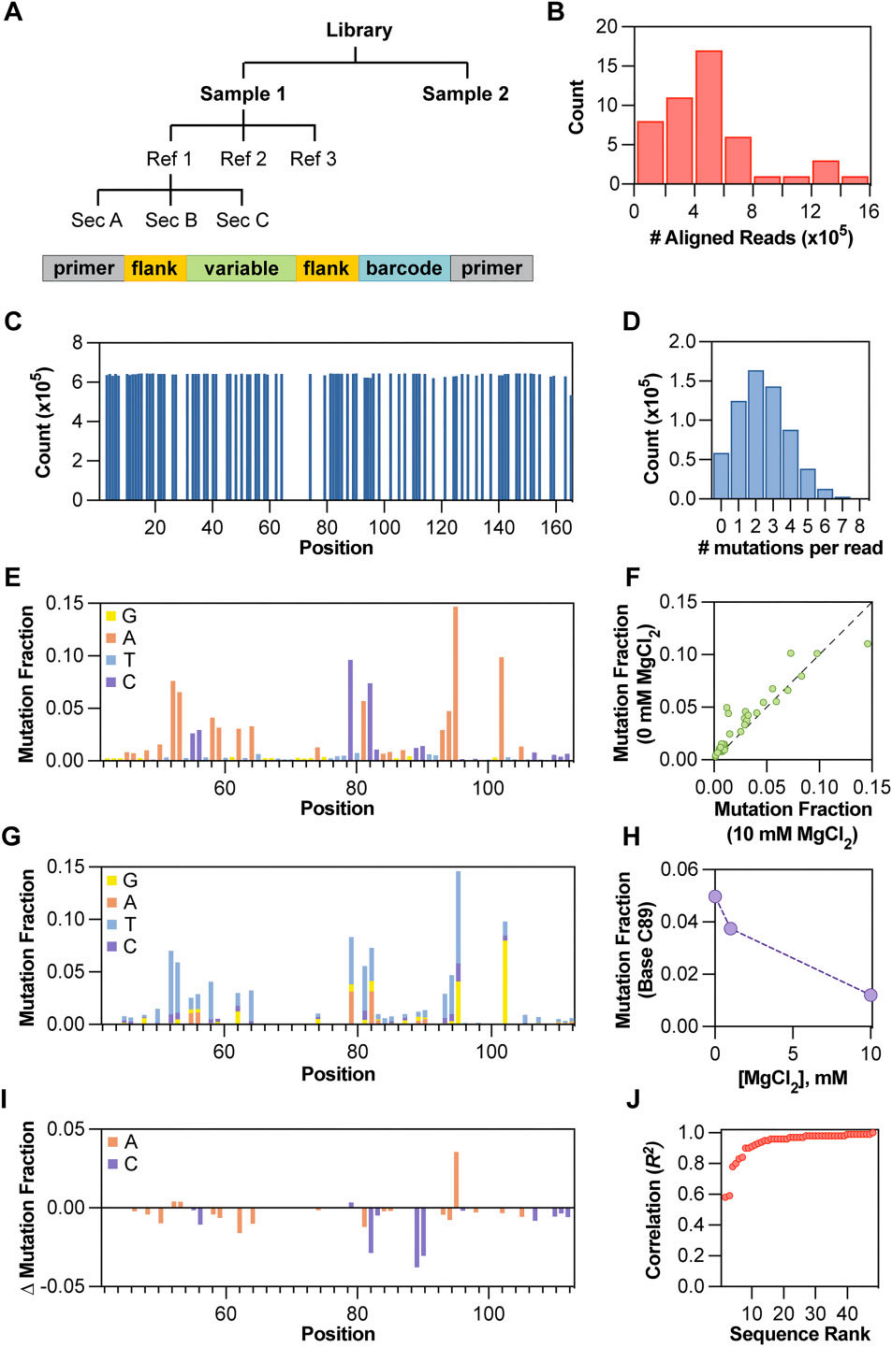
Examples of plot types available in SEISMICgraph. (**A**) Hierarchical data structure used in SEISMICgraph. For this library, each sample was separated into its reference sequences, which in turn were divided into sections. The references were structured to have common primers for library-specific amplification, a reference-specific barcode, variable-length flanking sequences to ensure all library members are of the same size, and a variable region that contains the RNA sequence of interest (see the “Materials and methods” section and Supplementary data). (**B**) # Aligned reads/reference as frequency distribution for one sample of a designed library of known RNA structures. (**C**–**I**) Example plots for one reference in the library where the variable region of interest is the *add* riboswitch (*V. vulnificus*) aptamer domain. (C) Base coverage for entire amplicon (showing only As and Cs, section = full). (D) Mutation per read per reference shows the frequency distribution of mutations per read in bins. (E) Mutation fraction for the variable section. Bases are colored by their identity in the reference sequence, where A is orange, C is purple, T is light blue, and G is yellow. (F) Compare mutation profiles for two samples with no MgCl_2_ or 10 mM MgCl_2_. Each green circle represents one reactive A or C base (*N* = 33; Pearson *R* = 0.93, RMSE = 0.011). The black dashed line represents the line of identity predicted for the null model of no difference between the samples (x = y). (G) Mutation fraction identity for the variable section. Each stacked bar represents the fraction mutated to that base, where orange represents bases mutated to A, purple represents bases mutated to C, light blue represents bases mutated to T, and yellow represents bases mutated to G. (H) Experimental variable across samples. The mutation fractions from three samples (0, 1, and 10 mM MgCl_2_) are shown for one base (C at position 89). (I) Mutation fraction delta comparing the variable section of two samples (0 and 10 mM MgCl_2_) for only A and C bases. The change in mutation fraction here is calculated as sample 1 (10 mM MgCl_2_) − sample 2 (0 mM MgCl_2_). Bases are colored by identity in the reference sequence, where A is orange and C is purple. (**J**) Correlation by references between samples. The Pearson correlations (*R*^2^) for all the members of the library were calculated by comparing the 0 versus 10 mM MgCl_2_ samples, where each red circle is an individual reference sequence.

Under Plot selections, the user can select from several plots provided by SEISMICgraph. The Supplementary data lists the plots that can be generated, and Fig. 2 and Supplementary Fig. S2 highlight examples of each plot type for short and long RNA sequences, respectively. The full details for additional plotting options are given in the “Plot selection options” section in Supplementary data.

Plot viewer displays the rendered plot. The plot can be downloaded as an image or interactive HTML page. Additionally, the data can be downloaded as a CSV file for further analysis or to plot in ways not available in SEISMICgraph.

A full tutorial for using SEISMICgraph is provided as a Supplementary document.

### Using SEISMICgraph to visualize riboswitches and pseudouridylation’s impact on function

**Figure 3.**
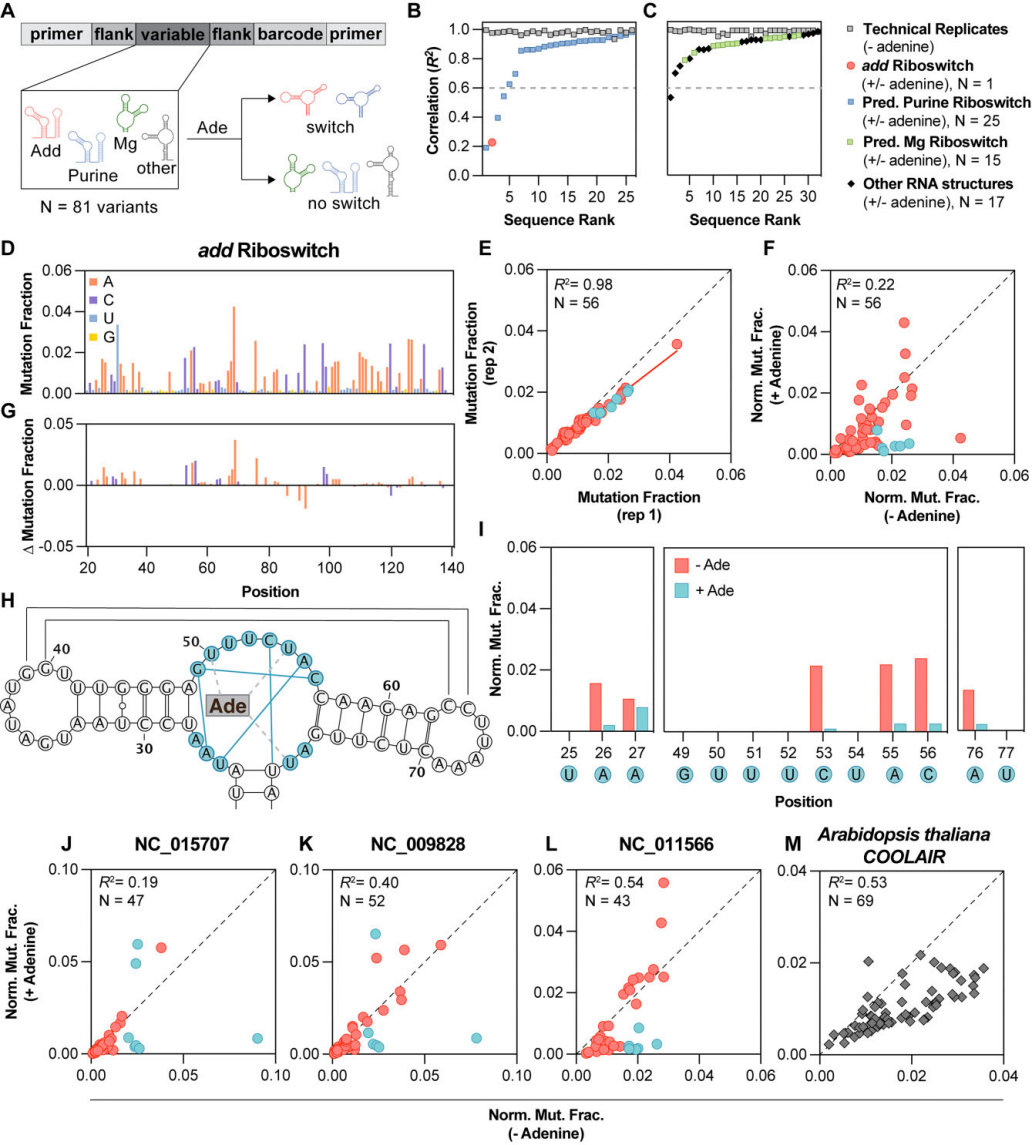
Experimental identification of adenine-responsive RNAs. (**A**) Library design for the riboswitch library. In this experiment the structure is probed with DMS in the presence or absence of adenine (Ade) to assess which structures undergo a conformation change. (**B**, **C**) Ranked correlation of references compared to each other in technical replicates (gray squares) or in samples with and without 5 mM adenine. (**D**) Mutation fraction at each position of the riboswitch section. (**E**) Comparison of mutation fraction at each position of two technical replicates where each red circle is an A or C base (*N* = 56). Turquoise circles depict A and C bases in the aptamer binding domain as in panel (H). The Pearson correlation (*R^2^*) was calculated in SEISMICgraph. The black dashed line represents the line of identity, and the red solid line represents the best-fit line from linear regression (slope = 0.77). (**F**) Comparison of the mutation fraction of samples in the presence and absence of 5 mM adenine. (**G**) Mutation fraction delta comparing samples in the presence and absence of 5 mM adenine, where the difference is calculated as sample 1 (with adenine) − sample 2 (without adenine). (**H**) Predicted secondary structure of the aptamer domain of the *add* riboswitch [25], where bases in the adenine binding site are depicted as turquoise circles. Hydrogen bond interactions enforced by binding between bases in the binding site are shown with turquoise solid lines [26]. Hydrogen bonds between the binding site and adenine are shown as gray dashed lines [26]. (**I**) Comparison of bases in the binding site in the absence (red bars) and presence (turquoise bars) of 5 mM adenine. (**J**–**L**) Putative purine riboswitches that undergo conformational change in response to adenine. Bases are colored as in panel (F). (**M**) A sequence from *Arabidopsis thaliana* that has a significant change in correlation upon addition of adenine. For panels (F), (G), and (I)–(M), samples were normalized as described in the Supplementary data. See also Supplementary Figs S2, S4, and S5.

We used SEISMICgraph to visualize and analyze DMS data for a designed library of RNA sequences to evaluate several predicted riboswitches, characterize several RNAs with well-established structures, and determine the effects of base modifications on the structure and function of these RNAs. Our library consisted of 81 RNAs, categorized as follows: 30 computationally-predicted purine riboswitches from various prokaryotic species [23], 29 predicted Mg^2+^ riboswitches, and 22 other RNAs with well-characterized structures (Fig. 3A). A complete list of sequences and their identities is provided in the Supplementary Table S1. We chose to use relatively short sequences in the library to enable high read depth and reduce amplification bias. For more information refer to the “Library design” section in Materials and Methods. Additionally, we provide detailed reports for all data and constructs in the Supplementary Dataset. We strongly encourage practitioners of chemical modification experiments to use and publish analogous summaries to make these studies more transparent and accessible to the scientific community.

#### Identification of purine-responsive riboswitches

The designed library was transcribed *in vitro* and folded in the presence or absence of adenine. The samples were then subjected to DMS treatment followed by library-specific RT-PCR [19] to detect conformational changes due to ligand binding (Fig. 3A–C).

As a positive control, we first evaluated the adenosine deaminase (*add*) riboswitch from *V. vulnificus*, which is known to alter its conformation upon adenine binding [19, 25]. We demonstrated reproducibility in the DMS signal through two technical replicates, as shown in the Mutation Fraction plots of those replicates (Fig. 3D and Supplementary Dataset “U1_Vibrio_vulnificus_0 and U2_Vibrio_vulnificus_0”) and the compare mutation profiles scatterplot (Fig. 3E, Pearson *R*² = 0.98 and Supplementary Fig. S2B, Pearson *R*² = 0.99). SEISMICgraph enables downstream analysis and visualization of mutation profiles, including normalization, signal difference calculations, and correlation analysis. In contrast, a comparison of the *add* riboswitch with and without adenine revealed differences, indicated by a much lower Pearson correlation coefficient relative to the replicates (Pearson *R*² = 0.22; Fig. 3F). These differences were also apparent from inspection of the mutation fraction across the sequence (Fig. 3D compared to Supplementary Fig. S3A) and the difference plot between these conditions (Fig. 3G). We also observed this adenine-dependent change in signal when analyzing our dataset using RNA Framework (Supplementary Fig. S4) and ShapeMapper2 (Supplementary Fig. S5), with both outputs visualized with SEISMICgraph.

The ability to download data from SEISMICgraph for further analysis allows users to closely examine single nucleotide changes under different conditions. For the *add* riboswitch, we identified individual bases within the aptamer binding domain that substantially decreased in DMS reactivity when adenine was present (Fig. 3H and I). The bases that were protected from DMS methylation upon ligand binding align with previous studies [25] and the hydrogen-bonding interactions enforced by adenine binding in the X-ray crystal structure of the ligand-bound state [26]. Such detailed analyses can enhance our understanding of the structural transformations induced by ligand binding. Future experiments can expand this approach to study the effect of other metabolites as well as study these riboswitches in cells.

Next, we analyzed our entire RNA library using SEISMICgraph to compare the DMS reactivity in the presence and absence of adenine (Fig. 3A). To provide an overall metric for adenine-dependent changes, we compared Pearson *R*² values between replicates and between experimental conditions (Fig. 3B and C). Three putative purine riboswitches, in addition to the established *add* riboswitch control, exhibited markedly lower *R*² values (<0.6) compared to all other sequences (Fig. 3B and C). Thus, DMS-MaPseq chemical probing can be used to identify candidate riboswitches. In all three putative riboswitches, the adenine binding pocket is highly conserved (Supplementary Fig. S7B–E), and a decrease in DMS reactivity is observed at these bases, akin to what is seen in the *add* riboswitch (Fig. 3J–L). The putative riboswitch from *Shewanella piezotolerans* is found upstream of a gene encoding adenosine deaminase (Fig. 3L), and the other two putative riboswitches (from *Thermotoga thermarum* and *Thermotoga lettingae*) are located upstream of operons involved in *de novo* IMP biosynthesis (Fig. 3J and K). Thus, it is reasonable that the three identified riboswitches are authentic, although direct functional tests in cells are needed. Equally interesting are the RNAs with sequence homology (Supplementary Fig. S6) that did not change conformation upon addition of adenine. These might adopt different, functional structures in cells or have evolved to bind different ligands or carry out different functions.

Surprisingly, one RNA lacking homology to adenine riboswitches also appeared to adopt an altered conformation in the presence of adenine. A short sequence from the *A. thaliana* gene COOLAIR also appeared to be responsive to adenine (Fig. 3M and Supplementary Fig. S8). While the 5′ domain of exon 1 from which this sequence was taken has several internal loops that might serve as a binding pocket for adenine [27], there is no observable pattern akin to that seen in the purine riboswitches. The shift in conformation may represent an accidental binding event [28] or an underlying yet-to-be-identified riboswitch function in a eukaryotic RNA. Regardless, the ability of RNAs to associate with nucleobases and other small ligands has implications for evolution in an RNA world and for potential modern-day RNA functions [28, 29].

The “other” RNA structures included in the library primarily serve as controls, allowing us to confirm that these structures can fold similarly to previous reports and to ensure that the addition of adenine does not affect the chemical probing experiment in the absence of binding. For example, FSE from HIV has a mutational profile consistent with stem-loop formation in the absence of adenine (Supplementary Fig. S9A and B). This pattern remains unchanged in the presence of adenine (Supplementary Fig. S9C and D).

Putative Mg-sensing riboswitch structures have a motif or junction that could act as the binding pocket for Mg^2+^, similar to those found in known and putative purine riboswitches. We determined if sequences predicted to shift in response to Mg^2+^ also change conformations in the presence of adenine. Of the 15 sequences that passed quality control filters, none showed an observable conformational change (all Pearson *R*^2^ > 0.6, Fig. 3C) at the working conditions (300 mM Na-cacodylate, 6 mM MgCl_2_). For example, sequence NC_0009792 appears to be folded in the absence of adenine (Supplementary Fig. S10A and B), and its conformation does not change in the presence of adenine (Supplementary Fig. S10C and D). Future experiments could measure the thermodynamics of folding by varying the Mg^2+^ concentration or temperature, using SEISMICgraph to analyze the results.

#### Pseudouridylation prevents conformational switching upon the addition of adenine

**Figure 4.**
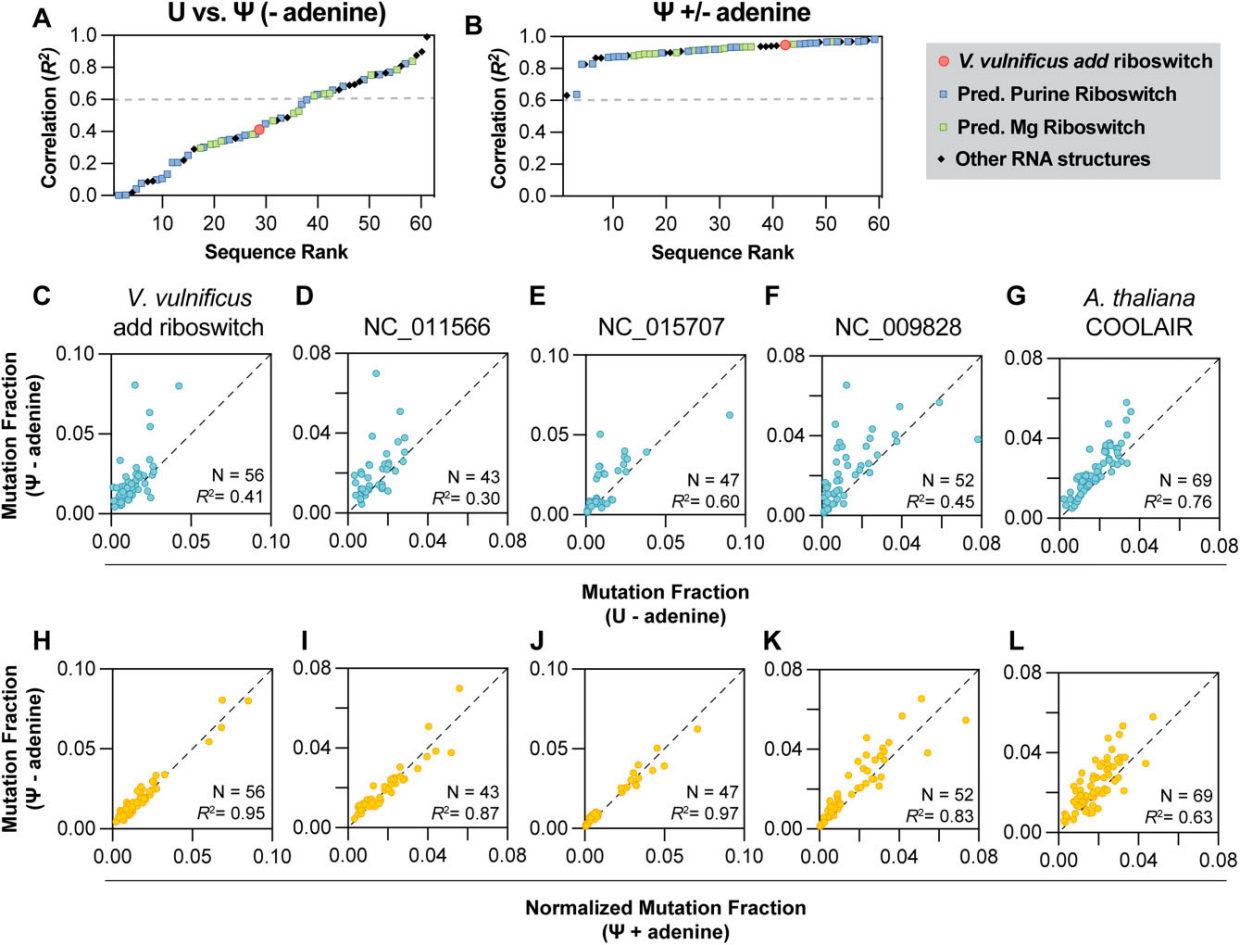
Effects of Pseudouridylation on RNA structure and riboswitching. (**A**) Ranked correlation of references compared to each other in samples containing all uridines or all pseudouridines. (**B**) Ranked correlation of references compared to each other in samples containing all pseudouridines in the presence and absence of 5 mM adenine. Each symbol represents one reference. (**C**–**G**) Comparison of the DMS signal of adenine-responsive sequences from Fig. 3 transcribed either with all uridines or all pseudouridines in the absence of adenine. Each turquoise circle represents one A or C base in the variable section. Neither sample was normalized. (H–L) Comparison of the DMS signal of adenine-responsive sequences from Fig. 3 transcribed with all pseudouridines in the presence or absence of adenine. Each yellow circle represents one A or C base in the variable section. The sample 2 (Ψ + adenine) is normalized to sample 1 (Ψ − adenine) as described in Supplementary data. For panels (C)–(L), N represents the number of A and C bases. The Pearson *R*^2^ was calculated in SEISMICgraph.

Pseudouridine (Ψ) and *N*^1^-methylpseudouridine (m1Ψ) are naturally occurring post-transcriptional modifications used widely in RNA therapeutics due to their ability to evade immune recognition and increase translation [30]. Since pseudouridylation has previously been shown to exert both stabilizing and destabilizing effects on RNA structure [31–33], we examined the functional consequences these modifications would have on the purine riboswitches analyzed earlier. The procedure was repeated as before, but with uridine (U) replaced with either Ψ or m1Ψ during *in vitro* transcription of the library (Fig. 4 and Supplementary Figs S11 and S12).

Incorporation of Ψ resulted in structural changes in more than half of the library sequences passing quality control filters (37 of 60; Fig. 4A). These changes indicate broad structural transformations, with decreasing levels of change highlighted in Supplementary Fig. S8. Similar DMS patterns, indicative of structural rearrangements, were also observed in sequences modified with m1Ψ, detailed in Supplementary Fig. S12B (17 of 59). These structural effects reflect the chemical properties of Ψ and m1Ψ, which both contain a C5-C1′ bond that augments base-pairing, base-stacking, and duplex stability, likely reducing the energy cost of non-native structures [31, 34]. While the DMS reactivities in Ψ and m1Ψ modifications generally demonstrated some correlation, indicating similarities in their structural impacts, the extent of these similarities varied depending on the sequence (Supplementary Fig. S12C and D). This variability highlights that Ψ and m1Ψ exhibit distinct behaviors in different sequences, likely due to the fact that Ψ has an extra hydrogen bond donor at N1, which is absent in m1Ψ, and is therefore capable of wobble base-pairing with G, U, or C in a duplex [35]. The differences are further suggested by the even more drastic structural changes seen upon incorporation of Ψ compared to m1Ψ. Notably, the introduction of either modification prevented conformational changes in all four adenine-sensing riboswitches and the COOLAIR structure (Fig. 4C–L). Given that Ψ has been shown to exert context-dependent destabilizing effects in functional RNAs, as in the *Escherichia coli* 23S rRNA and the neomycin-sensing riboswitch [32, 33], modifications in the U-rich aptamer domain of the *add* riboswitch would likely also disrupt tertiary contacts crucial for native folding, thereby eliminating adenine-sensing and functional switching of the pseudouridylated riboswitches.

## Discussion

We developed SEISMICgraph to streamline and standardize the analysis of chemical probing data. This tool provides various plotting and filtering options that enable rapid assessment and quantitative evaluation of experimental samples. It facilitates a broad overview by allowing researchers to examine multiple gene references simultaneously while also supporting detailed analysis by focusing on specific sequences. Additionally, SEISMICgraph allows for fast and thorough comparisons across different samples, experimental conditions, and sequences, making it a versatile and efficient resource for RNA structure research (Fig. 2 and Supplementary Figs S2 and S13).

We confirmed three key findings using SEISMICgraph. First, we validated the structural changes in a well-documented adenine riboswitch. Second, we characterized the structures of a library of 81 sequences, including 30 of putative purine riboswitches, and found that three exhibit changes in response to adenine. Third, we demonstrated that introducing base modifications results in defects in structural switching. Notably, Ψ and m1Ψ modifications prevent the adenine-sensing function of the *add* riboswitch and the three other purine-responsive riboswitches.

These findings are particularly relevant in the context of RNA-based therapeutics, such as messenger RNA vaccines [36], where modifications like pseudouridylation are commonly employed to evade host immune detection [37–39]. As the use of RNA in biotechnology continues to expand to include structured, functional RNAs [40], it becomes increasingly important to assess how widespread application of such modifications may affect their structural integrity and therapeutic efficacy. Chemical probing techniques such as DMS-MaPseq and SHAPE, combined with the visualization and analytical capabilities of SEISMICgraph, allow precise identification of when and where these structural disruptions occur, offering valuable insights for the rational design of functional, modified RNA therapeutics.

## Supplementary Material

gkaf701_Supplemental_Files

## Data Availability

SEISMICgraph is available at https://seismicrna.org. Code is available at https://rouskinlab.github.io/seismic-graph/ or at FigShare (Python package: https://figshare.com/articles/software/seismic-graph_python_package/27111424?file=49427674; Webapp codebase: https://figshare.com/articles/software/SEISMICgraph_Website_Codebase/27111421?file=49427671). All data collected in this study can be found at the Sequence Read Archive, accession PRJNA1165154.

## Notes

Present address: Department of Chemistry, Texas A&M University, College Station, TX 77843, United States

Present address: Department of Microbiology, Harvard School of Medicine, Boston, MA 02115, United States

Present address: Department of Chemistry, Texas A&M University, College Station, TX 77843, United States
