# Supplementary material for: SEISMICgraph: a web-based tool for RNA structure data visualization
Source: Nucleic Acids Res. 2025 Jul 31;53(14):gkaf701. doi: 10.1093/nar/gkaf701 (PMC12311782; doi:10.1093/nar/gkaf701)
Supplement: gkaf701_Supplemental_Files [file gkaf701_supplemental_files.zip › SupplementaryFile_SEISMICgraphTutorial.pdf]

Using SEISMICgraph

a web-based tool for RNA structure visualization

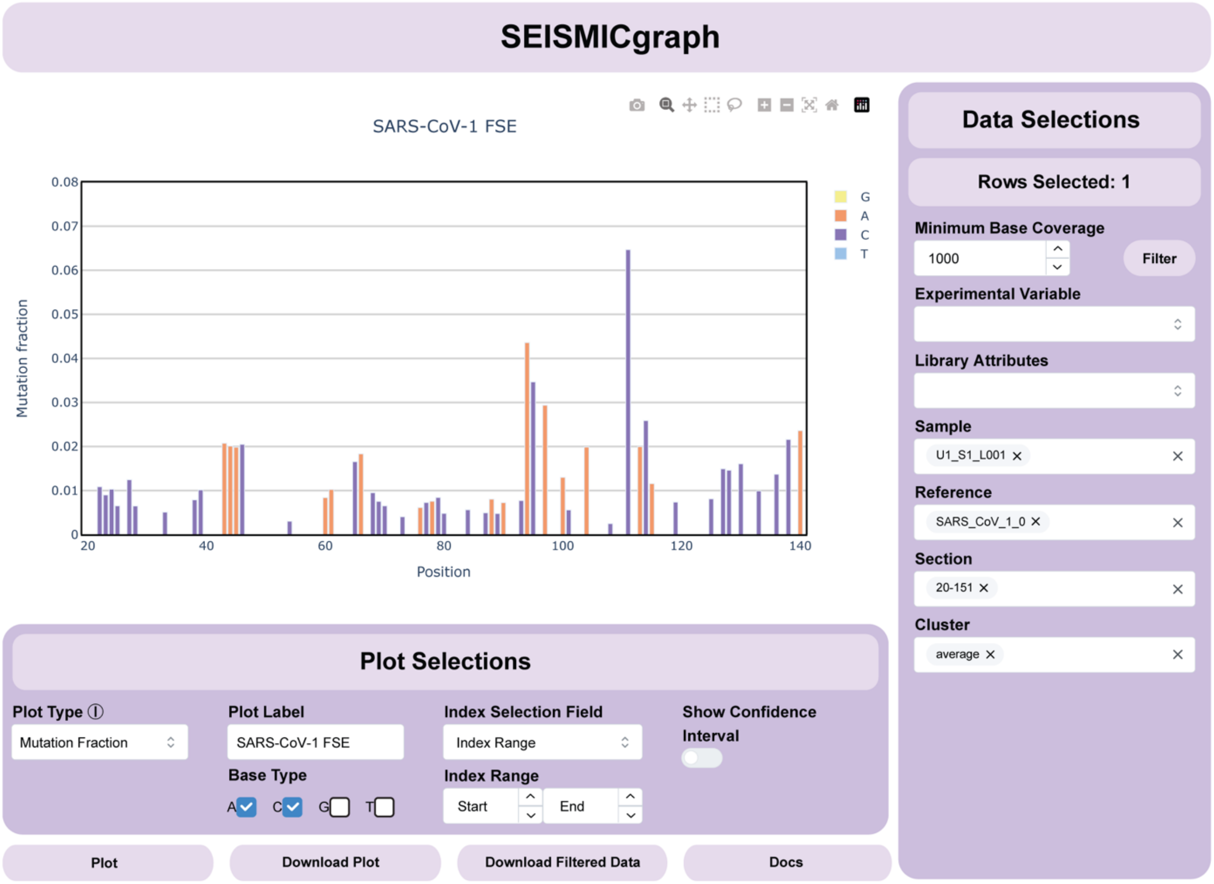

Contents

1. Introduction **3**

1.1. Accessing SEISMICgraph 4

1.2. Sample dataset 4

2. Working with a single sample **5**

2.1. Upload a dataset 5

2.2. Make data selections 6

2.3. Make plot selections 6

2.4. Downloading plot and the associated data 7

2.5. Other plot types that use one row selected 8

2.6. One pager 11

2.7. Other plot types with one sample 12

3. Working with multiple samples **13**

3.1. Uploading multiple samples 13

3.2. Make data selections 13

3.3. Make plot selections 13

3.4. Other plot types that require multiple samples 15

4. Conclusion **17**

1 Introduction

Welcome to the SEISMICgraph tutorial! This guide is designed for first-time users and aims to provide a clear, step-by-step introduction to the functionality and features of SEISMICgraph, a powerful interactive platform for visualizing and interpreting RNA structure probing data.

SEISMICgraph is part of the SEISMIC (Systematic Evaluation and Integration of Structural Measurements to Interpret Change) RNA framework, which enables researchers to explore RNA structure dynamics in response to cellular context, experimental conditions, or perturbations. The platform presents an intuitive graphical interface that integrates multiple structural readouts, making it easier to identify biologically meaningful structural changes across datasets.

In this tutorial, we will:

- Familiarize you with the layout and core features of the SEISMICgraph interface.
- Walk you through each type of plot and its biological significance.
- Highlight how SEISMICgraph can support data exploration, pattern recognition, and biological insight.

Whether you're a chemical biologist, RNA researcher, or computational biologist, this tutorial will help you get started with SEISMICgraph and understand how it can support your exploration of RNA structural landscapes.

Let's dive in!

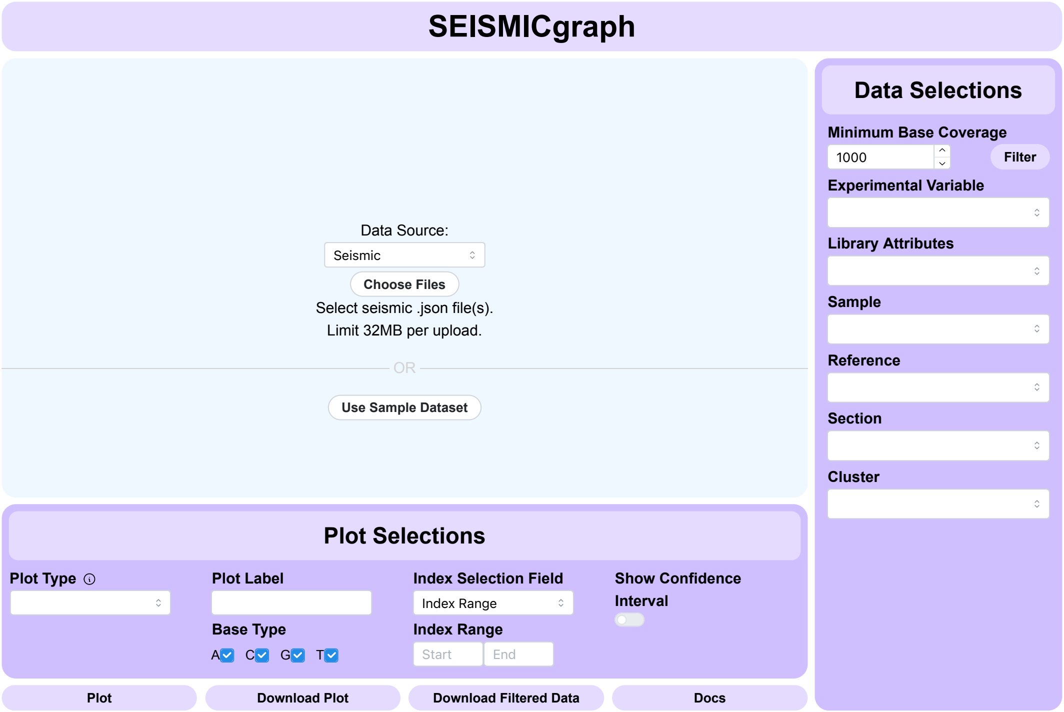

1.1 Accessing SEISMICgraph

Before beginning the tutorial, open the SEISMICgraph website by navigating to www.seismicrna.org in your web browser.

Once the page loads, you'll see the main interface, which is divided into several key areas:

- **Display Window (Center):** This is where all the plots will appear once you load data. It's the main area for visualizing results.
- **Data Selection Panel (Right Side):** Use this panel to choose the sample, reference, and experiment condition you want to explore.
- **Plot Selection Panel (Bottom):** This section allows you to choose which types of plots to generate.

Below the plot selection panel, you'll also find several important buttons:

- **Plot Data:** Click this after selecting your data and plot types to generate the visualizations.
- **Download Plot:** Save a copy of the currently displayed plot.
- **Download Filtered Data:** Export the underlying data used for the plot.
- **Source Docs:** Access related documentation or metadata for the dataset.

Take a moment to get familiar with the layout—it will make the rest of the tutorial easier to follow.

1.2 Sample Dataset

For this tutorial, we'll be using a sample dataset from the paper *"SEISMICgraph: a web-based tool for RNA structure data visualization."* The dataset consists of a curated library of 81 short RNAs that were in vitro transcribed and structurally probed using DMS-MaPseq under various conditions. The library includes three main groups: 30 computationally predicted purine riboswitches from diverse prokaryotic species, 29 predicted Mg^{2+}-responsive riboswitches, and 22 additional RNAs with well-characterized secondary structures.

Each RNA was transcribed using one of three nucleotides—uridine (U), pseudouridine (P), or N1-methylpseudouridine (N)—in addition to A, C, and G to assess the impact of modified nucleotides on RNA structure. Structural probing was performed using DMS-MaPseq both in the absence and presence of 5 mM adenine (e.g., comparing U vs. UA), allowing for the detection of ligand-induced structural changes. Each condition was performed in duplicate (e.g., comparing U1 vs. U2) to ensure reproducibility.

This dataset captures a broad range of structural behaviors, including both ligand-dependent and ligand-independent changes, making it an ideal system for learning how to use SEISMICgraph to visualize and interpret RNA structural dynamics.

2 Working with a single sample

In this section, you will learn the basic functions of SEISMICgraph. We will start with loading a sample dataset, selecting the data and plot type, and rendering images. This section of the tutorial uses sample "U1_S1_L001" as an example.

2.1 Upload a dataset

The first step is to load our sample dataset:

Access SEISMICgraph at (www.seismicrna.org). In plot display panel, choose the **Data Source** (a). SEISMICgraph supports data outputs from SEISMIC, ShapeMapper2, and RNA Framework.

1. After selecting the data source, click ***Choose Files*** (b) to select the files to upload. This will open a file window to your computer. You may choose to select one or more datasets depending upon how many samples or conditions you would like to compare. After selected the file(s) you would like to plot, hit ***upload*** button to add them to SEISMICgraph.

2. Alternatively, you may choose to explore the functions of SEISMICgraph by hitting the ***Use Sample Dataset*** (c) button. This tutorial will use the sample dataset to generate basic plot types.

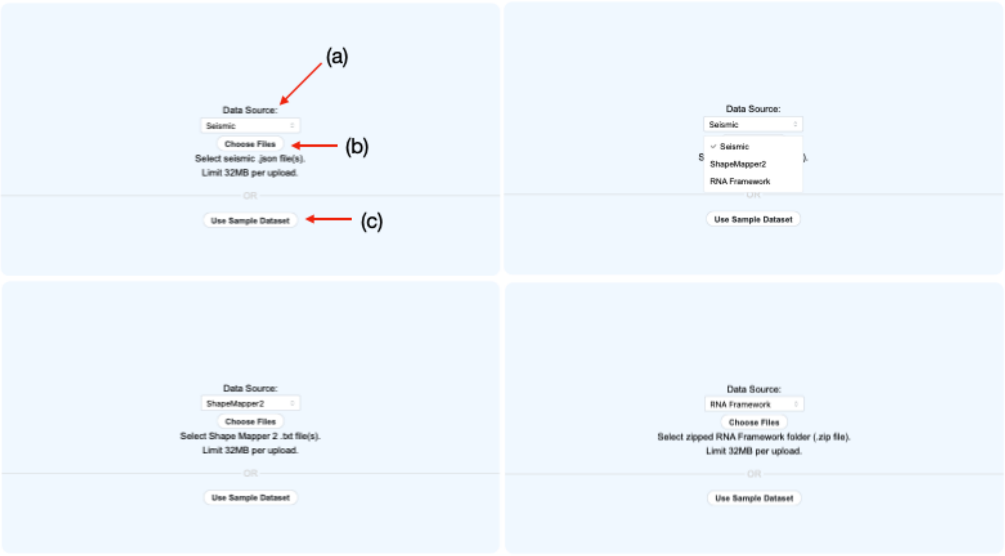

You will now notice that the **Data Selections** pane displays a number of **Rows Selected**. For the sample dataset this number is 1401.

2.2 Make data selections

To plot the chemical probing data, we must first select the data that will go into the plot. We will make data selections from the pane and the number of rows selected should indicate the available data for the plot. To make a simple plot of the **Mutation Fraction** per position, we will need to select one sample and one reference sequence.

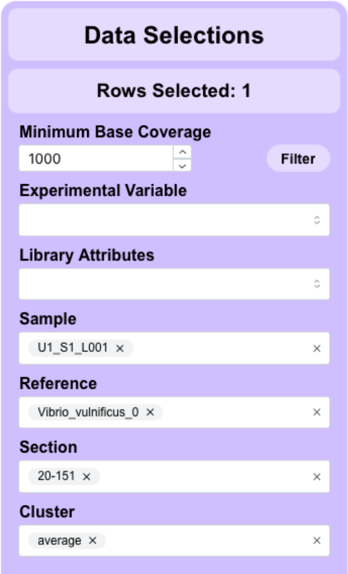

1. Set the **Minimum Base Coverage**: This is set to 1000 reads per base by default, but you may want to make this more stringent based on your research question or signal size.

2. Select one sample from the **Sample** dropdown. For this example, we will select only the “U1_S1_L001” sample. The number of **Rows Selected** should decrease to 97.

3. Select one reference from the **Reference** dropdown. For this example, we will select only the “Vibrio_vulnificus_0” reference. The number of **Rows Selected** should decrease to 1.

For this simple, plot we have omitted selections for **Experimental Variable** and **Library Attributes**, but we will comment on these in later sections. You will also notice that **Section** and **Cluster** auto-populate here because there is only one section and cluster available in the sample for this reference.

2.3 Make plot selections

Now that we have identified selected a single row to plot, we must set the plot selections. First, we will select “Mutation Fraction” under the **Plot Type**. This will yield a bar graph of the mutation fraction per position. To plot the data, simply hit the ***Plot*** button on the bottom left corner of the screen. Additionally, the plot can be labeled with a header by typing in the **Plot Label** box. Bases can be omitted by de-selecting them under **Base Type**. The **Index Selection Field** allows you to indicate how the data should be indexed and what numbers should start and end the index. Finally, **Show Confidence Interval** allows you to calculate the 95% CI of the mutation fraction. This CI reflects both the signal size and number of reads.

The **Plot Viewer** in the center of the screen is interactive—the axes can be changed to capture individual features of the data; certain position can be zoomed in on by drawing a box around them; certain bases can be de-selected by clicking on their label in the legend; etc.

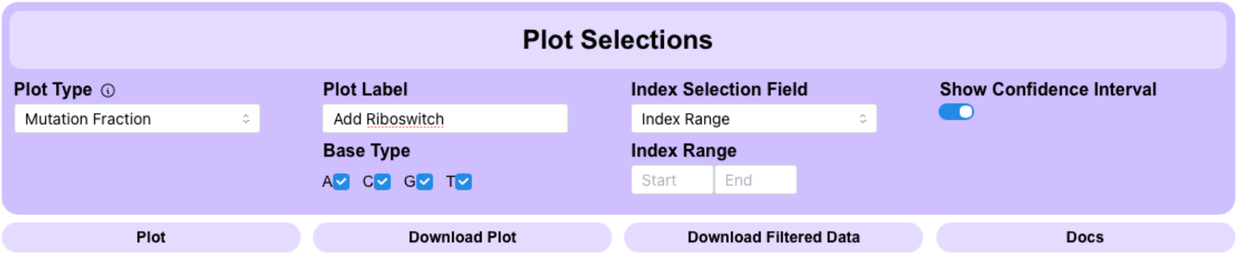

Your plot should look something like this following the steps above:

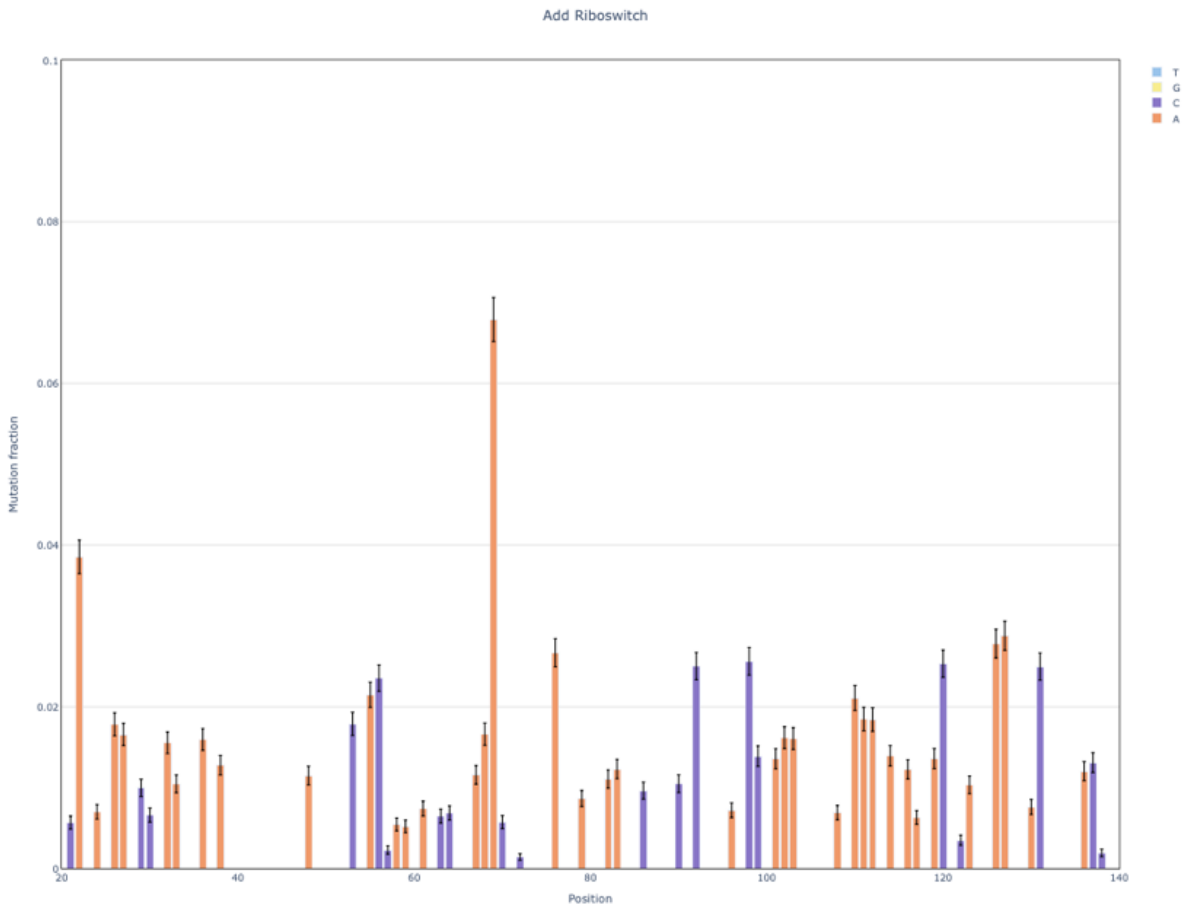

2.4 Downloading a plot and the associated data

To download the plot, we can use the ***Download Plot*** button on the bottom of the screen or the camera icon in the top right corner of the plot viewer. To download the filtered data frame, use the ***Download Filtered Data*** button.

2.5 Other plot types that use one row selected

There are several plots that require that only one row be selected examples of these include:

Base Coverage

A bar plot depicting the number of reads at each position for a given reference and section.

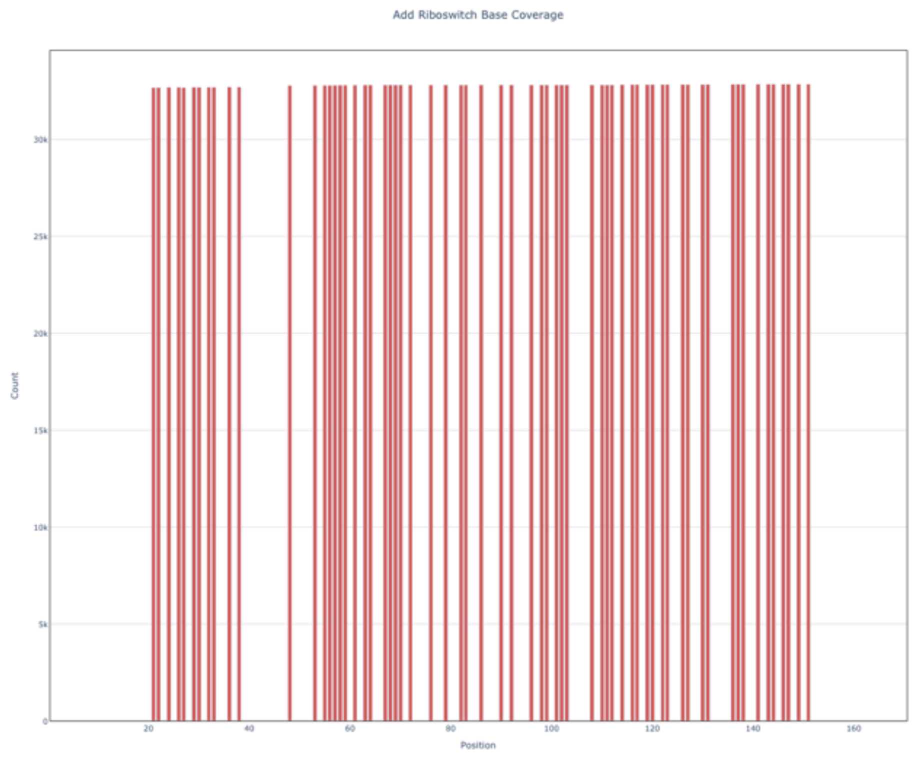

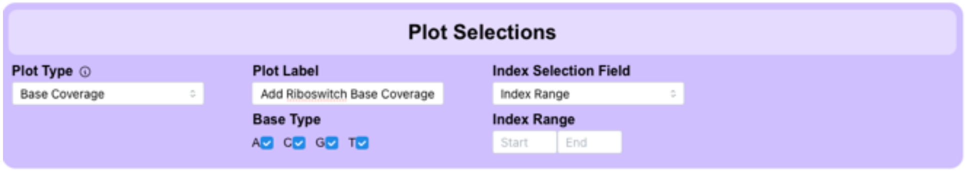

Mutation Fraction Identity

A stacked bar plot, quantifying the number of mutations at a given position and identifying the mutation from either DMS-MaPseq or SHAPE-MaPseq after reverse transcription.

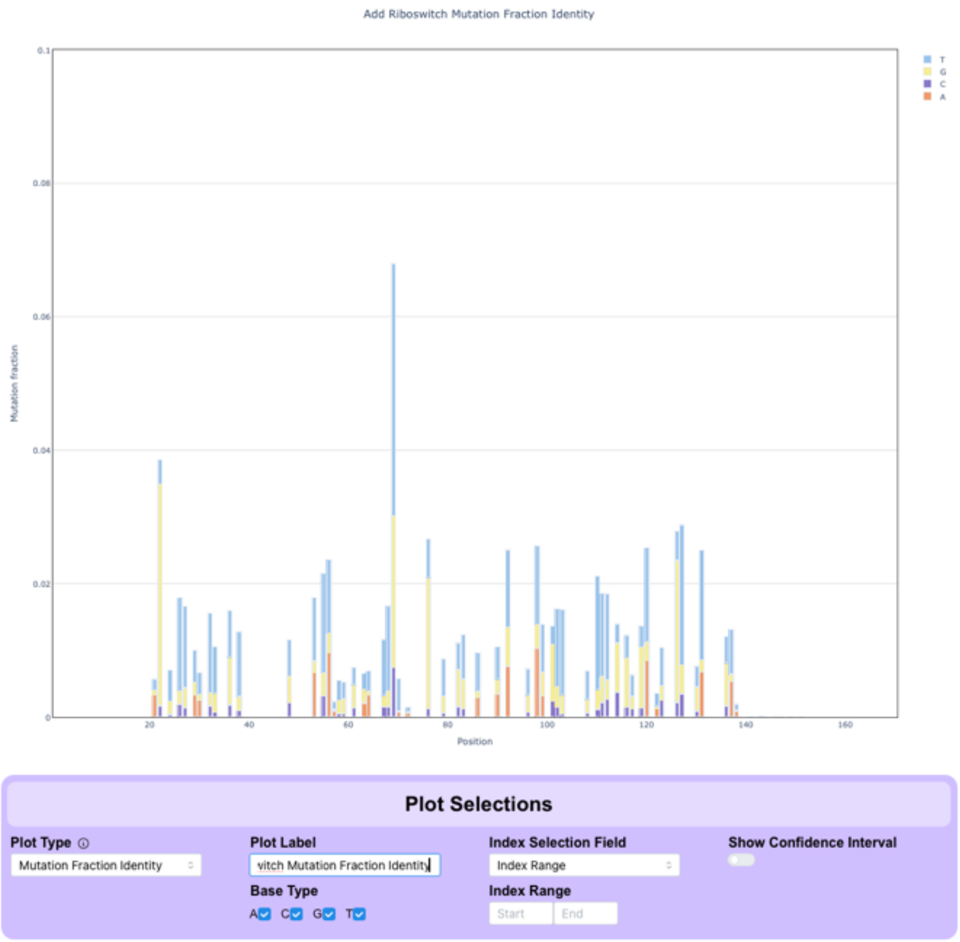

Mutations per Read per Reference

A histogram showing the frequency distribution of mutations per read in bins.

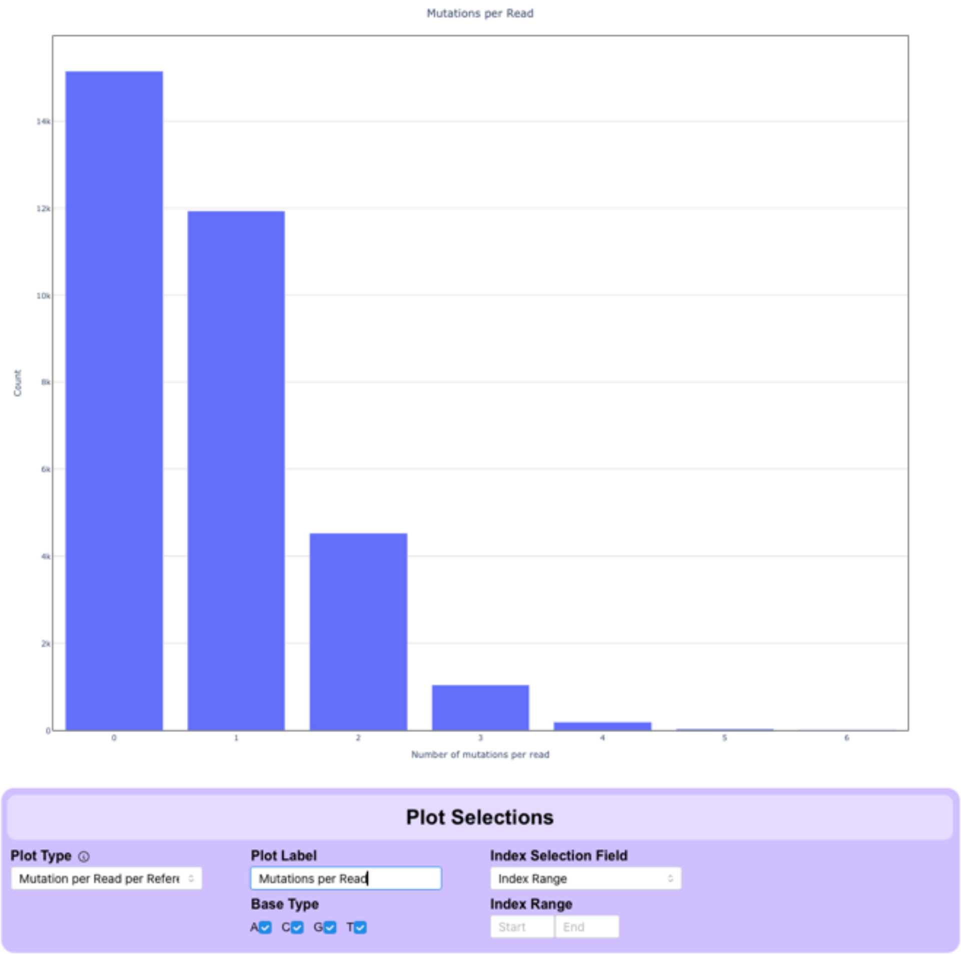

2.6 One pager

The plot option "**one pager**" can be used to compare all of the plots for one reference in one sample at the same time. Using this plot function will generate a new pop-up window. All of the associated plots (e.g., base coverage, mutations per read, mutation fraction, and mutation fraction identity) are interactive. Additionally, the "one-pager" prints the relevant metadata for the experiment. This feature is great for compiling all of the associated plots together at one time.

U1_S1_L001 - Vibrio_vulnificus_0

Experimental info table

Sample	U1_S1_L001
Reference	Vibrio_vulnificus_0
User	Grant
Date	09-30-2023
Temperature	37° C
System	sodium cacodylate (300 mM)
Probe	DMS
Concentration	5% v/v
Metabolite	Control
Modification	Control

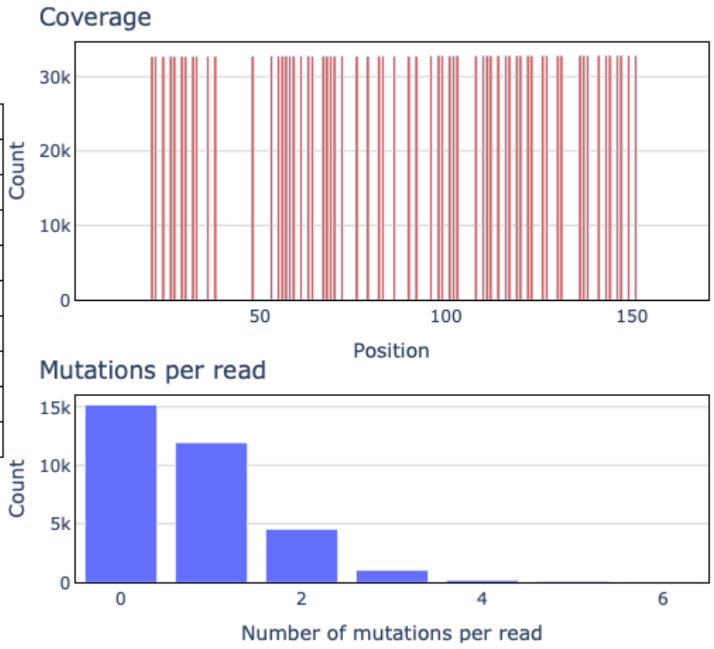

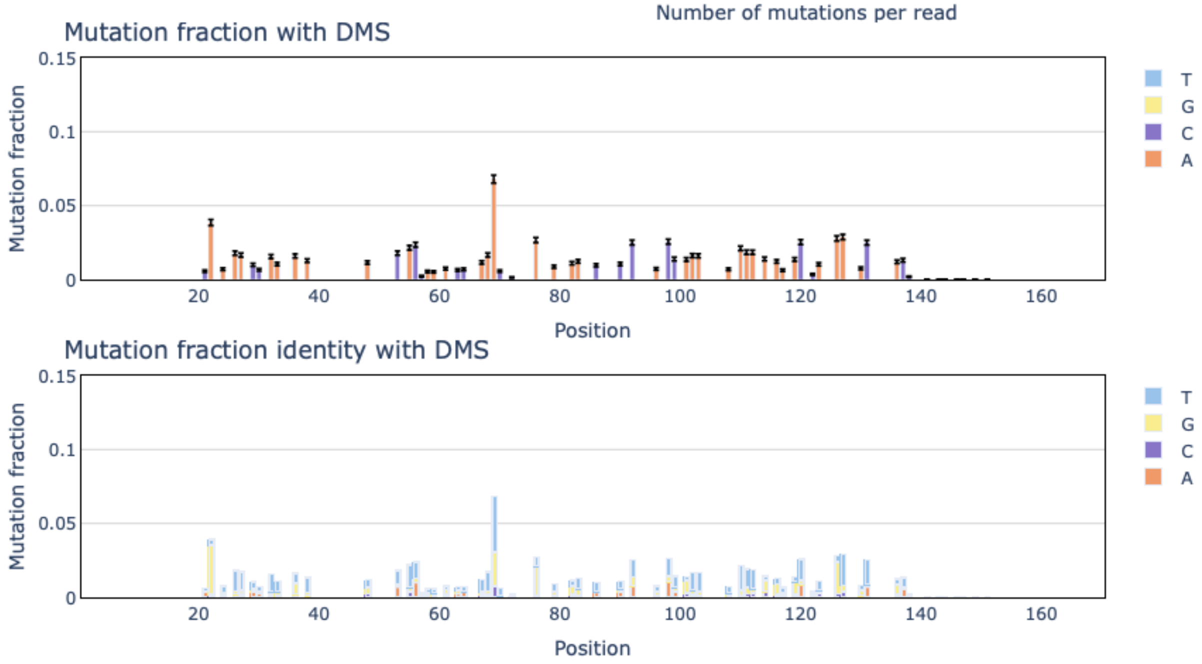

2.7 Other plots that require one sample

The Plot Type "# Aligned Reads / Reference as Freq. Dist." can be generated for a single sample without selecting one reference. This plot makes a frequency distribution for the number of reads aligned to each reference in a sample. This can be useful for library experiments where you would like to have equal distribution of reads across all the reference in the library.

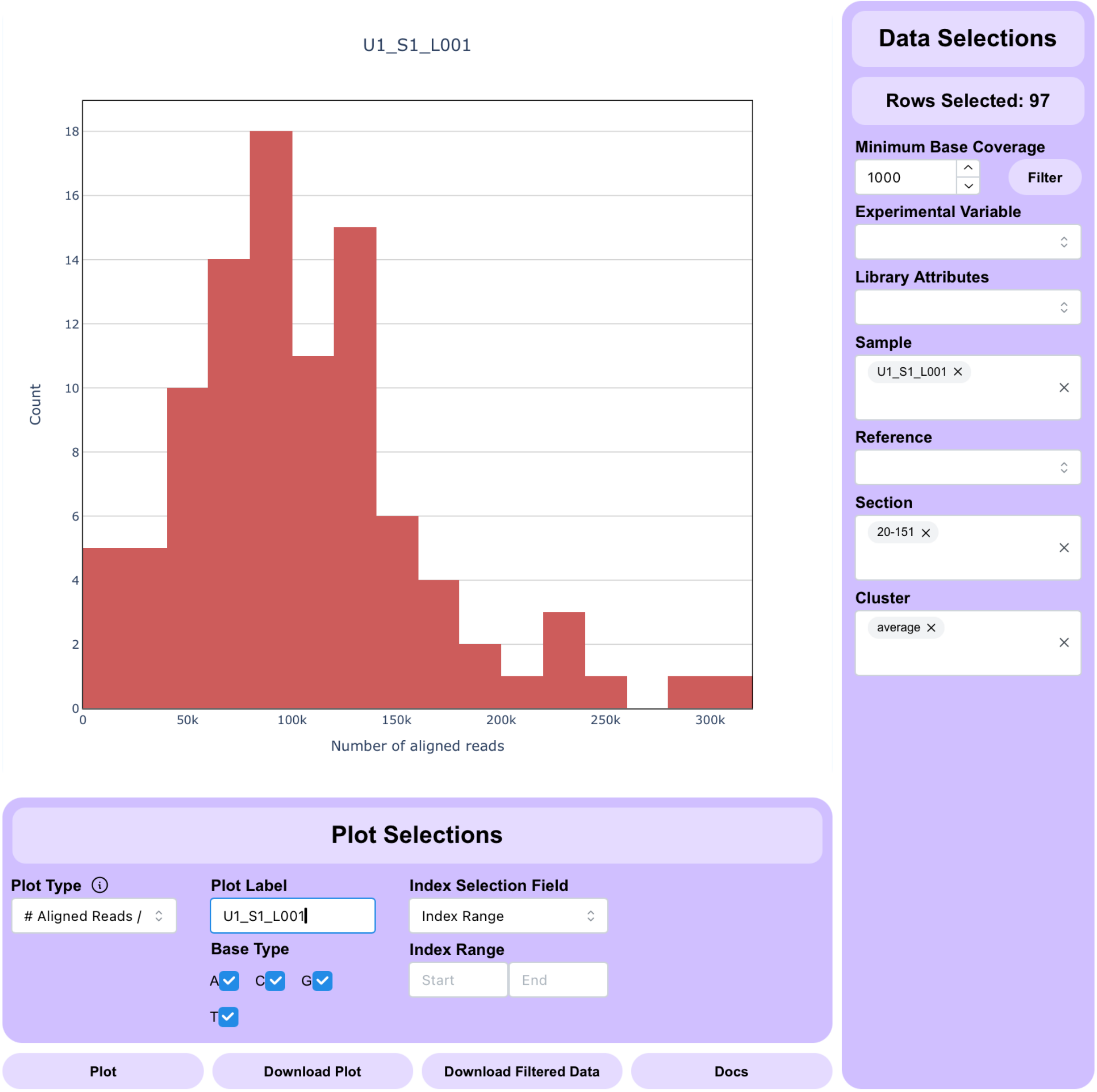

3 Working with multiple samples

In this section, you will learn how to generate plots that compare more than one sample at a time using SEISMICgraph.

Here, we define a sample as one experimental condition paired with a single replicate. In practical terms, this typically corresponds to one set of FASTQ files from a sequencing run. For example, "U1" refers to a sample where the RNA was transcribed with uridine, treated under a specific condition (e.g., without adenine), and sequenced once. Its biological replicate would be "U2," representing the same condition but from an independent experiment.

When comparing multiple samples, you might examine:

- Differences between replicates to assess reproducibility (e.g., U1 vs. U2)
- The effect of ligand binding (e.g., U vs. UA)
- The impact of nucleotide modifications (e.g., U vs. P vs. N)
- Or combinations of the above.

As you work through this section, you'll learn how to select and load multiple samples, configure the plot settings, and interpret the resulting visualizations.

3.1 Uploading multiple samples

From the www.seismicgraph.org homepage, you can elect to upload more than one sample json file. Using the same steps as section 1, select 2 or more samples before hitting ***upload***. The process is possible with data processed using ShapeMapper2. In the example dataset, there are many samples uploaded at once for comparison.

Note: only 1 sample can be visualized at a time with data processed using RNA Framework.

3.2 Make data selections

For this example, we will start by comparing one reference in two biological replicates. We will set the **Minimum Base Coverage** as 1000 as described in section 2. Under **Sample**, we will select "U1_S1_L001" and "U2_S2_L001" which are biological replicates of the same library. Finally, we will again select "Vibrio_vulnificus_0" as our **Reference**. This process should decrease the number of **Rows Selected** to 2.

3.3 Make plot Selections

Under **Plot Type**, we will select "Compare Mutation Profiles." This creates an x,y-scatter plot comparing the two samples. We can again label the plot, omit bases, and change the index as needed. You will notice two additional options here. Under Pearson Threshold, REMIND ME WHAT THIS MEANS. For this example, we will leave the Pearson Threshold set to none. You can also choose to normalize one dataset to the other by activated the ***Normalize*** button. Finally, we will press the ***Plot*** button to plot the data. This should yield a scatter plot with the mutation fraction

of U1_S1_L001 on the x-axis and the mutation fraction of U2_S2_L001 on the y-axis. Each blue circle represents one reactive A or C base. The green line represents the line of identity (x=y). The red line represents the line of best fit from a linear regression. The equation for line is reported in the plot viewer as well as the Pearson (R) correlation, the Coefficient of Determination (R^2) and the root-mean-square-error (RMSE). The equation for the linear regression is also reported.

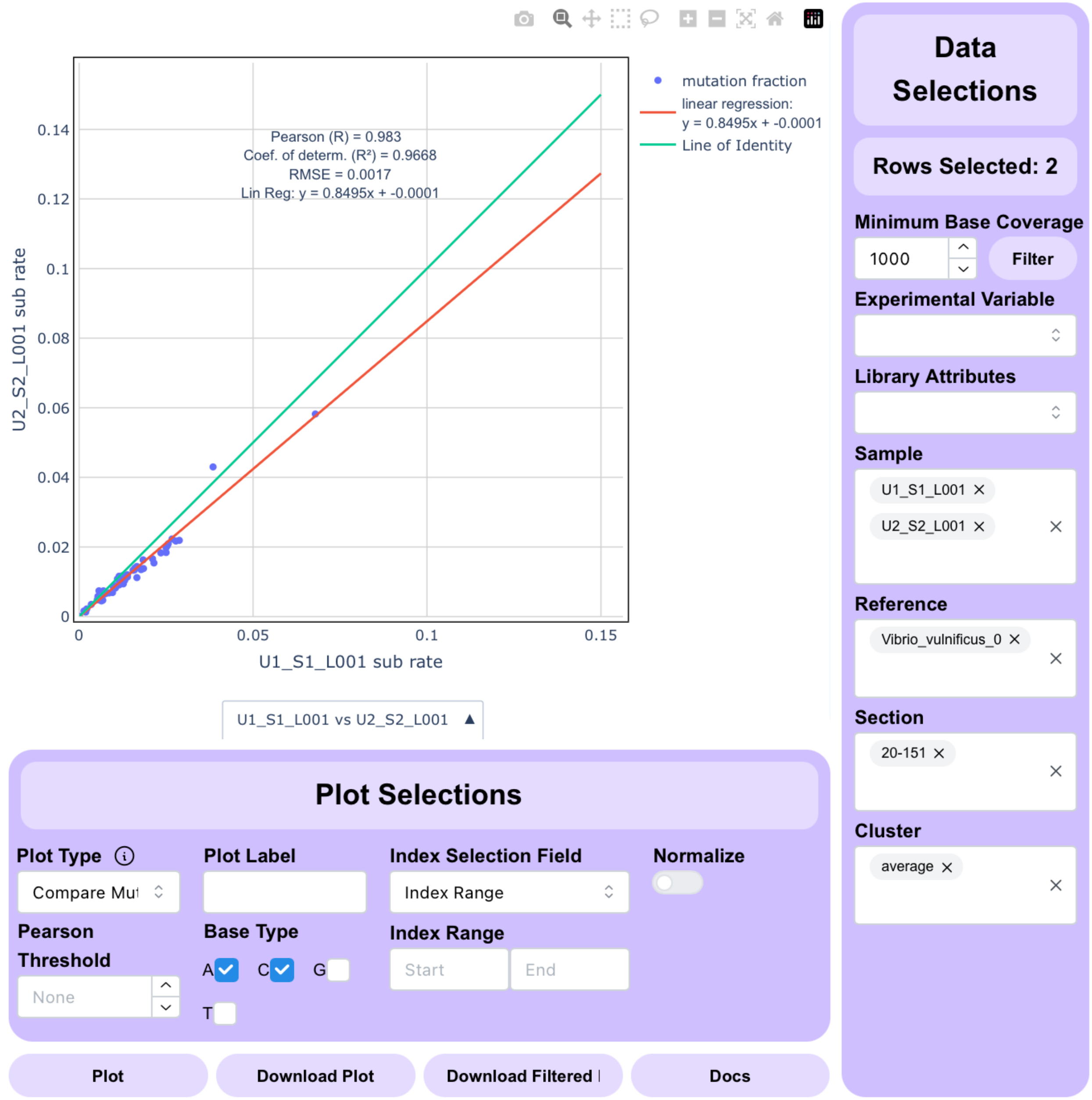

3.4 Other plot types that require multiple samples

Correlation by Reference Between Samples

This plot can be used to calculate the Pearson Correlation between two references for each common reference. For this example, we will compare a sample run in the absence of ligand “U1_S1_L001” with a sample in the presence of ligand “UA1_S7_L001.” Here each blue circle represents an individual reference sequence.

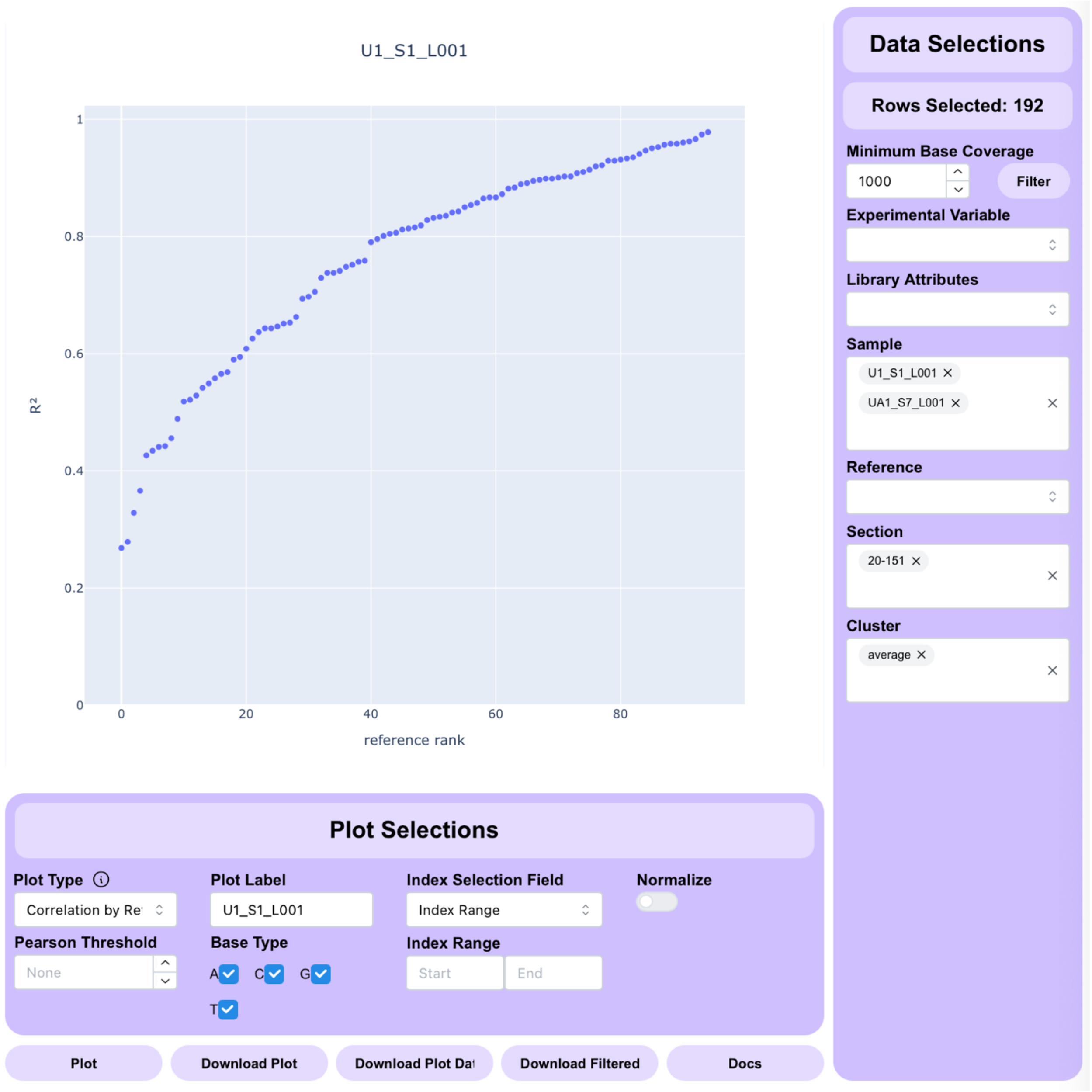

Mutation Fraction Delta

This plot can be used to calculate the difference in mutation fraction between two **Samples**. For this example, we will compare a sample run in the absence of ligand “U1_S1_L001” with a sample in the presence of ligand “UA1_S7_L001” for **Reference** “Vibrio_vulnificus_0.”

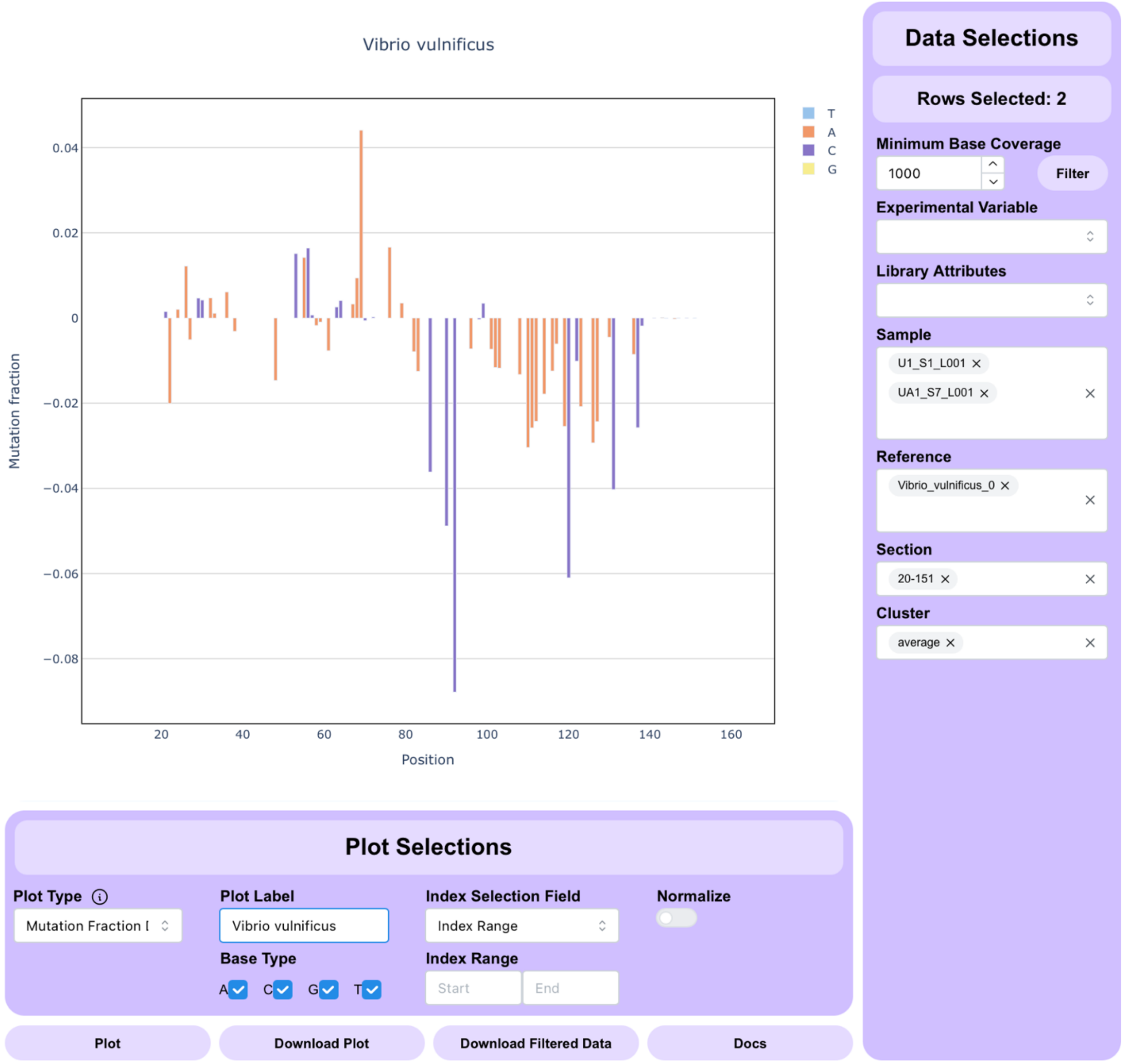

Experimental Variable Across Samples

Optionally, you may select the **Experimental Variable** you are interested in filtering for under Data Selections. Once you click inside of the selection box, the available options will appear. For example, selecting “DMS_concentration_pct_volume” filters for all the rows were the amount of dimethyl sulfate concentration (v/v%) was specified. Another box will appear below to allow you to select one or more concentrations of interest. This dataset only contains one DMS concentration (5% v/v), but you could select as many concentrations as needed.

To generate a plot comparing the change in experimental value for a given reference, select all the experimental variable and values you would like to compare and the reference in Data Selections. Under Plot Selections, select the Plot Type “Experimental Variable Across Samples.” You will also need to select the experimental variable from the drop-down menu in Plot Selections.

The Experimental Variable could be anything and is specified in the metadata provided to SEISMIC when processing the data.

4 Conclusion

You’ve now completed the SEISMICgraph tutorial and explored how to navigate the interface, interpret structural plots, and compare multiple conditions across a well-characterized RNA library. With these tools in hand, you’re ready to apply SEISMICgraph to your own experiments. Whether you're investigating the effects of ligands, RNA modifications, protein binding, or cellular context, SEISMICgraph provides a flexible and intuitive platform to visualize and interpret RNA structure probing data. We encourage you to upload your own datasets, explore structural patterns, and uncover new insights into RNA behavior. If you publish findings that use SEISMICgraph, please cite the associated paper—and feel free to share your feedback or feature requests to help improve the tool. Happy exploring!
